# Expression of particulate-form of Japanese encephalitis virus envelope protein in a stably transfected Drosophila cell line

**DOI:** 10.1186/1743-422X-4-17

**Published:** 2007-02-26

**Authors:** Fuquan Zhang, Wenyu Ma, Li Zhang, Marlen Aasa-Chapman, Hongyi Zhang

**Affiliations:** 1 Division of Biomedical Sciences, Faculty of Medicine, Imperial College London, London, UK; 2 Department of Microbiology, the Fourth Military Medical University, Xi'an, the People's Republic of China; 3 Institute for Animal Health, Pirbright Laboratory, Ash Road, Pirbright, Surrey UK; 4 HPA Clinical Microbiology and Public Health Laboratory, Addenbrooke's Hospital, Box 236, Hills Road, Cambridge CB2 2QW, UK

## Abstract

**Background:**

Japanese encephalitis virus (JEV), a member of the family *Flaviviridae*, is an important mosquito-borne human pathogen. Its envelope glycoprotein (E) is the major determinant of the pathogenicity and host immune responses. In the present study, we explored the feasibility of producing recombinant JEV E protein in the virus-free *Drosophila *expression system.

**Results:**

The coding sequence for the signal sequence of premembrane and E protein was cloned into the Drosophila expression vector pAc5.1/V5-His. A *Drosophila *cell line S2 was cotransfected with this construct as well as a plasmid providing hygromycin B resistance. A cell line expressing the JEV E protein was selected by immunofluoresence, confocal microscopy, and western blot analysis using three different monoclonal antibodies directed against JEV E protein. This cell line was stable in the yield of JEV E protein during two months *in vitro *maintenance in the presence of hygromycin B. The results showed that the recombinant E protein had an expected molecular weight of about 50 kilodalton, was immunoreactive with all three monoclonal antibodies, and found in both the cytoplasm and culture supernatant. Sucrose gradient ultracentrifugation analysis revealed that the secreted E protein product was in a particulate form. It migrated to the sucrose fraction with a density of 1.13 g/ml. Balb/c mice immunised with the sucrose fraction containing the E protein particles developed specific antibodies. These data show that functioning JEV E protein was expressed in the stable S2 cell line.

**Conclusion:**

The Drosophila expression system is a more convenient, cheaper and safer approach to the production of vaccine candidates and diagnostic reagents for JEV.

## Background

Japanese encephalitis virus (JEV) is a member of the genus *Flavivirus *in the family *flaviviridae*. It is the most common agent of viral encephalitis, causing an estimated 50,000 cases annually, of which 15,000 will die and up to 50% of survivors are left with severe neuropsychiatric sequelae [1,2]. Most cases occur in southern and eastern Asia, but the geographical area affected by JEV is expanding. Outbreaks have been reported in Saipan islands, Torres Straits islands and on Australia mainland in recent years [3-5]. Cases have also occurred among travellers and US servicemen to Asia [6,7]. In addition, related neurotropic flaviviruses are found across the globe; they share many virological, epidemiological, and clinical features.

The flavivirus viron contains an envelope glycoprotein (E), a membrane protein (M) and a capsid protein (C). These three structural proteins are synthesized in the order of C, M and E from the 5' half of a single long open reading frame of the flavivirus genome. The M protein is found in infected cells as a glycosylated precursor, called premembrane protein (preM). The preM and E proteins appear to be released from the nascent polyprotein following cotranslational cleavage by signal peptidases. Late in viron maturation, preM is cleaved to M, presumably by a cellular protease located in the secretary pathway, and M appears to be the predominant species present in extracellular virus particles although some uncleaved preM is also present [8]. Flavivirus-infected cells release not only infectious virons but also non-infectious subviral membrane particles containing the M and E proteins but no C protein or viral RNA, known as slowly sedimenting hemagglutinin particles [9]. Similar particles can be produced using various eukaryotic expression systems [10-16]. For JEV, such particles were produced in mammalian cells infected with recombinant poxviruses encoding the signal sequence of preM, preM and E proteins, and were designated as the subviral extracellular particles (EPs). They are membrane vesicles of 20 nm in diameter containing JEV preM/M and E proteins embedded in the lipid bilayer, and showed similar behaviour to the slowly sedimenting hemagglutinin particles released from JEV-infected cells on sucrose density gradients [10,17]. Mice immunised with EPs were protected from lethal JEV infection [11]. In addition, the JEV EPs were also found in plasmid-based mammalian cell expression system. The recombinant plasmid was tested as a DNA vaccine candidate against JEV: it elicited immune response in mice [18,19]. Partially purified JEV EPs were used as standard antigens for serodiagnosis of JEV infection [20]. JEV preM and E proteins were expressed in Sf9 insect cells infected with recombinant baculovirus, and the intracellular E protein was shown to be protective in mice against lethal JEV challenges [21,22]. It is not known whether EPs were formed or not in this system.

Despite of the successful expression of flavivirus E proteins and formation of subviral EPs in mammalian cells or baculovirus-insect cell system, the disadvantages related with these expression systems make it difficult to produce and purify EPs in a large scale: the culture of mammalian cells is expensive; virus-based expression is transient and the maintenance or scale-up of virus stock requires a considerable and dedicated effort; viral proteases and cell lysate can cause degradation of the desired proteins and it is difficult to separate desired EPs from the recombinant virus particles.

To address these problems, the nonlytic, virus-free Drosophila Expression System (DES) was employed in this study as an alternative approach to produce JEV E protein and EPs to be used as a vaccine candidate and diagnostic reagent. The DES utilises a cell line derived from *Drosophila melanogaster*, Schneider 2 (S2) cells, the genome of which has been completely sequenced [23], and a simple plasmid vector for the expression of heterologous proteins using either the metallthionein (MT) promoter [24,25] or the Actin 5C (Ac5) promoter [26]. S2 cells are easily maintained in loosely adherent or suspension culture at room temperature and do not require CO_2_. Many foreign signal sequences are functional in S2 cells and can be used to secrete proteins. This system has been used to produce many foreign proteins, including viral antigens [27,28]. In the current report, we described establishment of a stable S2 cell line producing JEV subviral EPs containing E protein and the characterisation of their antigenicity and immunogenicity.

## Results

### Sequence validation of recombinant expression vectors

The inserts of pAc/ZF-ZR and pAc/JEF-JER were sequenced. In comparison to pJEV1, the recombinant cosmid clone containing JEV cDNA and used as the template DNA (our unpublished data), no sequence differences were found. The cloning sites of all four constructs were confirmed to be the context as designed.

### Expression of JEV E protein in S2/ZF-ZR and S2/JEF-JER cells

**Figure 1:**
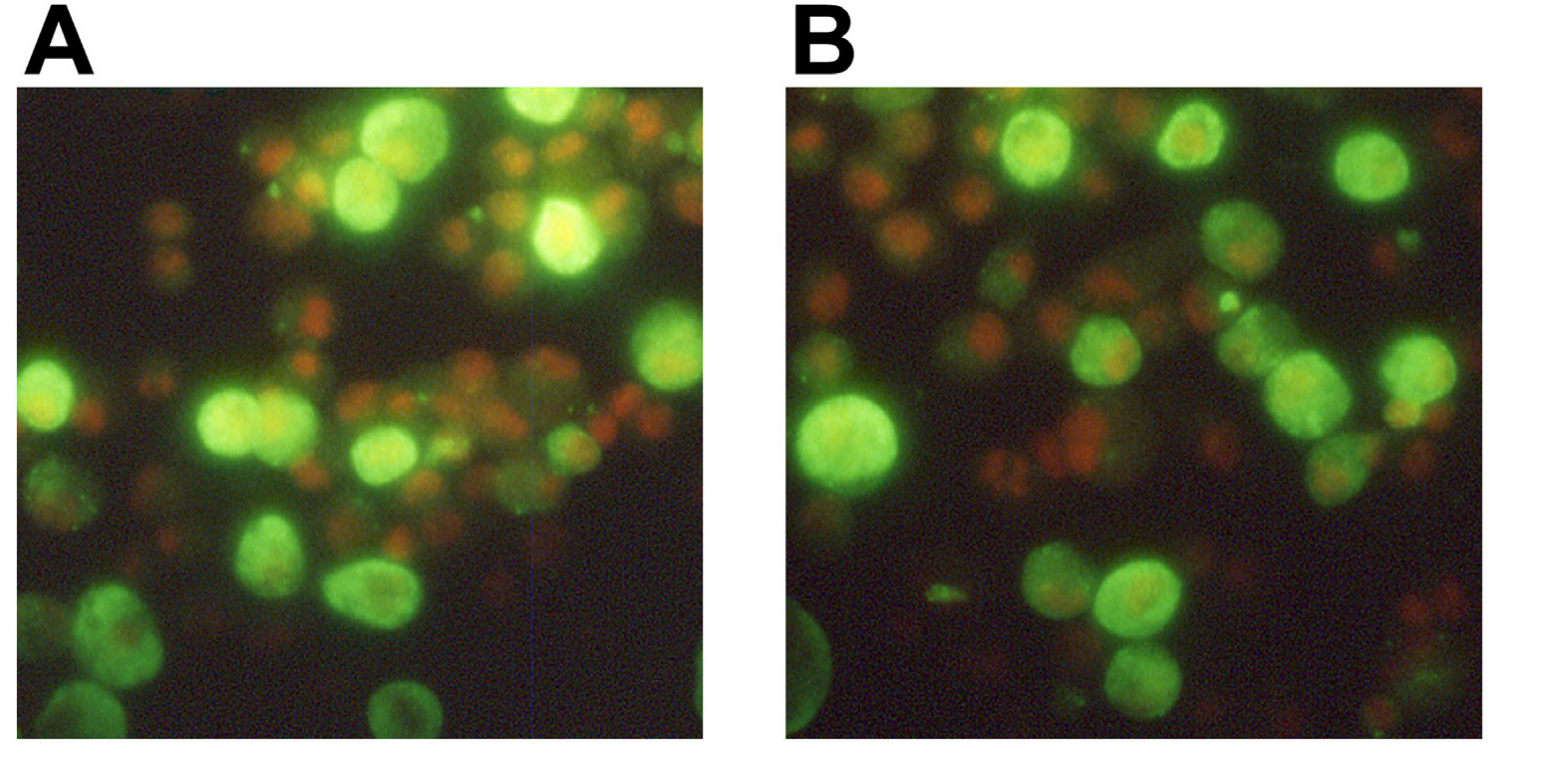
**Immunofluorescence analysis of S2/ZF-ZR and S2/JEF-JER cells with antibody 2F2**. S2 cells were transfected with pAc/ZF-ZR (frame A) or pAc/JEF-JER (frame B). Transfected cells were harvested in PBS and plated onto microscopic slides. After air drying, the cells were fixed in 10% formalin with 1% Triton X-100 at room temperature for 30 min. The cells were incubated subsequently with JEV E specific monoclonal antibody 2F2 and a fluorescein-conjugated secondary antibody (DAKO) at room temperature for 1 hr. Each incubation was followed by two washes in PBS. Finally the slides were mounted with VECTASHIELD Mounting Medium containing propidium iodide (Vector Laboratories) and viewed under an Olympus fluorescence microscope. The green fluorescence indicates the detection of JEV E proteins.

**Figure 2:**
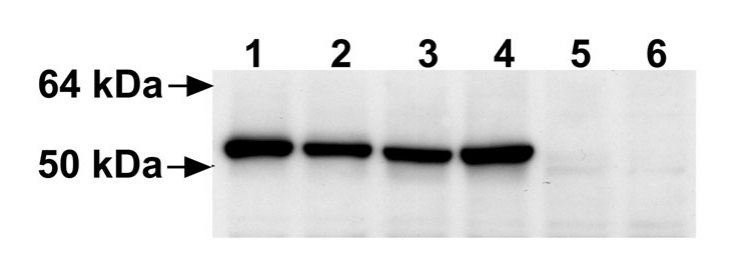
**Comparison of recombinant JEV E protein expressed in deferent cells by Western blot**. Cell lysate were resolved by 12% SDS-PAGE under non-reducing conditions. After electrophoresis proteins were transferred onto nitrocellulose membrane and probed with the antibody 2F2. ECL reagents were used to visualise protein bands. Lanes 1 to 6 are the cell lysate from Vero/ZF-ZR, Vero/JEF-JER, S2/ZF-ZR, S2/JEF-JER, Vero and S2 cells respectively.

Transfected S2 cells, either S2/ZF-ZR or S2/JEF-JER appeared normal morphologically and their growth was comparable with normal control S2 cells. Indirect immunofluorescence with monoclonal antibody (MAb) 2H4, 2F2 or mc3 revealed that the recombinant JEV E protein expressed in either cell line was accumulated in vesicle-like structures in the cytoplasma, and there was no cell surface staining (Fig. 1). This was confirmed by confocal microscopy (data not shown). There was no difference in staining patterns among these three antibodies. The recombinant JEV E protein expressed in S2/ZF-ZR, S2/JEF-JER or Vero cells transfected with pVAX/ZF-ZR or pVAX/JEF-JER was analysed by Western blot with antibody 2F2. They all had the same molecular weight of about 50 kDa as expected (Fig. 2).

**Figure 3:**
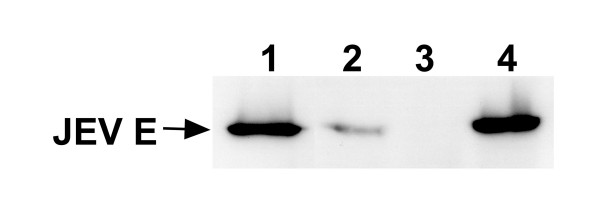
**Effect of preM signal sequence on the secretion of E protein from transfected S2 cells**. Cell lysate or culture supernatant was resolved by 12% SDS-PAGE under non-reducing conditions. After electrophoresis proteins were transferred onto nitrocellulose membrane and probed with the antibody 2F2. ECL reagents were used to visualise protein bands. Lanes 1 to 4 are S2/ZF-ZR cell lysate, S2/ZF-ZR culture medium, S2/JEF-JER culture medium and S2/JEF-JER cell lysate respectively.

To determine the effect of preM signal sequence on the secretion of E protein from transfected S2 cells, cell lysate and culture supernatant of S2/ZF-ZR or S2/JEF-JER, which has a 19-peptide or 9-peptide preM signal sequence respectively, were analysed by Western blot. The samples were standardised according to the cell number or culture density. S2/ZF-ZR and S2/JEF-JER had the similar expression level of intracellular form of E protein, while the extracellular E protein was only detected from the former culture supernatant by the Western blot (Fig. 3). Results show that all three MAb 2F2, 2H4 and mc3 were directed against conformational epitopes and disulfide bond dependent. They were reactive with the recombinant E protein expressed in either S2 or Vero cells only if the samples were prepared in the absence of the reducing agent such as β-mercaptoethanol or dithiothreitol.

### Characterisation of extracellular-form of JEV E protein expressed in S2/ZF-ZR cells

**Figure 4:**
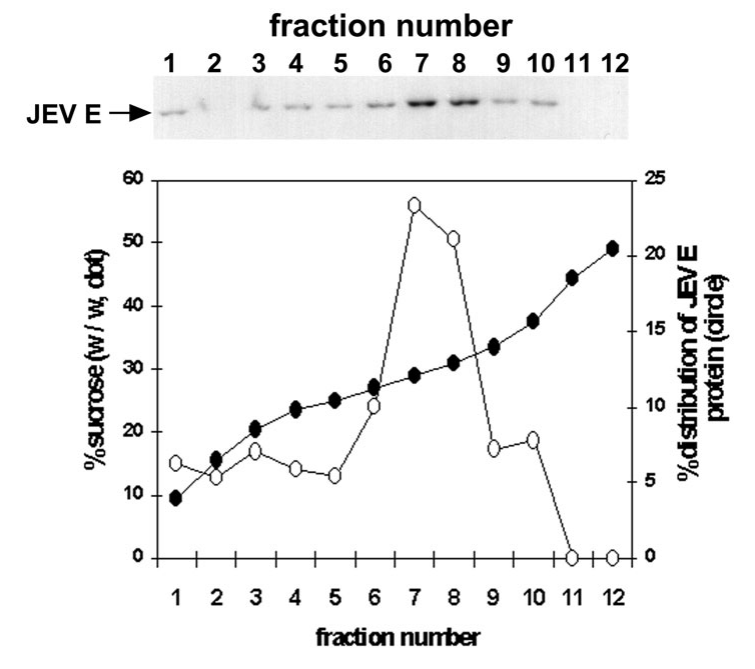
**Distribution of JEV E protein in the sucrose gradient after ultracentrifugation**. A, Western blot analysis of JEV E protein in the sucrose gradient fractions. B, density plot of the fractions and percentage distribution of JEV E proteins cross the fractions estimated by densitometry.

**Figure 5:**
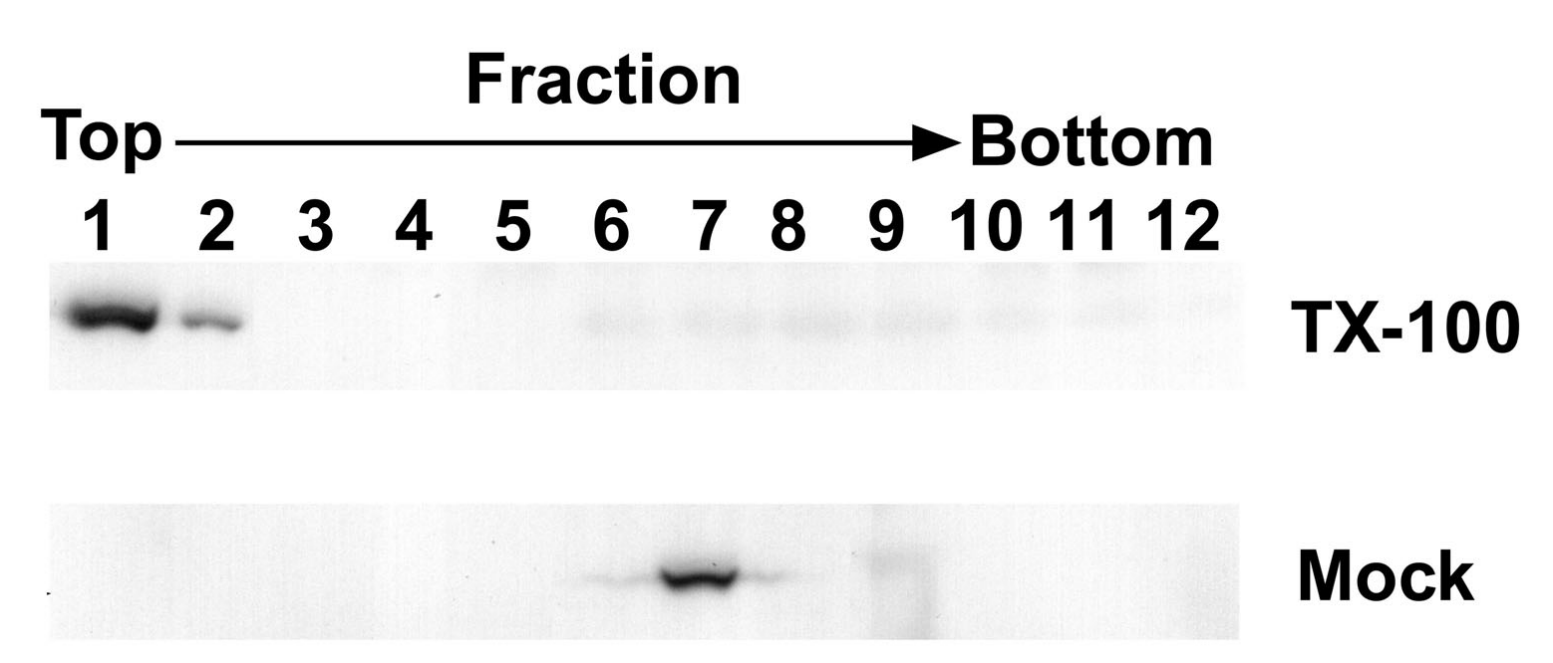
**Effect of TX100 on the EPs containing JEV E protein**. Sucrose centrifugation-purified extracellular particles were treated with Triton X-100 (final concentration of 1 mg/ml), and then applied again to a 10–60% continuous sucrose gradient (W/V) centrifugation. Fractions were collected and analysed by Western blot. A, Triton X-100 treated sample; B, mock treated sample.

The culture medium harvested from S2/ZF-ZR cells was centrifuged through a 10% (W/V) sucrose cushion, and Western blotting was used to determine the distribution of the extracellular E protein in the pellet, cushion or culture supernatant. The results demonstrated that about 90% of the extracellular E protein was sedimented through the sucrose cushion (data not shown). The pellet was resuspended and was further resolved by centrifugation through the 10–60% (W/V) sucrose gradient. Twelve fractions of 1 ml were collected from the top to bottom and analysed by Western blotting with antibody 2F2. The E protein was mainly found in fraction 7 and 8, which had an average density of 1.13 g/ml, but also detectable in others (Fig. 4). The Coomassie blue stained SDS-PAGE gel showed that E protein was the main protein component in fraction 7 and 8, and the average concentration of E protein in fraction 7 and 8 was estimated at 2 μg/ml by comparison with serial diluted bovine serum albumin (data not shown). To determine whether or not the extracellular form of E protein was associated with membrane vesicles, the sucrose gradient fraction containing the peak E protein (fraction 7) was diluted in PBS and treated with Triton X-100 at a final concentration of 1 mg/ml. Samples were applied again to the sucrose density gradient centrifugation as described in the Materials and Methods. As shown in Figure 5, following Triton X-100 treatment, the peak of E protein shifted upward in the gradient, becoming less dense. In contrast, the peak in mock-treated samples remained unchanged. These results indicate that most of the E protein in the culture medium was in a form of extracellular particles, in which the E protein was associated with membrane vesicles.

### Stability of the S2/ZF-ZR cell line

During a period of two months of in vitro maintenance in the presence of Hygromycin B, no morphological change was observed in the S2/ZF-ZR cell line. Cells were sampled and extracted at an interval of 2 weeks as described in the Methods and tested for JEV E protein by using Western blotting. All 5 samples had a similar expression level of JEV E protein as indicated by the Western blot band intensity (data not shown).

### Immune response in mice immunised with S2/ZF-ZR lysate and EPs

Balb/c mice (4 animal each group) were immunised with S2/ZF-ZR EPs, lysate or PBS buffer (mock) respectively to test the immunogenicity. Two weeks after second immunisation (boost), mouse sera were collected and tested for specific antibodies to JEV E protein by Western blot using the Vero/ZF-ZR cell lysate as the antigen. Serum samples diluted at 1:20 from 3 of 4 mice immunised with EPs were positive for JEV E specific antibodies, whereas all the mice inoculated with cell lysate or PBS were negative at a dilution of 1:10.

## Discussion

The signal-preM-E cassette of JEV genome has been well explored in both recombinant subunit vaccine and DNA vaccine studies. This is based on the fact that the E protein mediates receptor binding and membrane fusion and induces protective immunity [29], the maturation of JEV E protein requires cosynthesis of the preM protein [30] and the signal sequence of preM signal affects the secretion of the coexpressed E protein [17]. In the vaccinia-mammalian cell system, preM/M and E gene in this cassette were translated and processed correctly in terms of molecular mass, glycosylation and antigenicity compared to their natural counterparts. More importantly, the preM and E proteins expressed by recombinant vaccinia viruses could form slowly sedimenting hemagglutinin particle-like EPs, in which the conformation of E protein was similar to that in natural virons. Due to their good antigenicity and immunogenicity, such EPs may be very useful in both serological diagnosis and vaccination [11,20,31]. In addition to mammalian expression system, structural proteins of JEV and other flavivirus were also produced in recombinant baculovirus-infected insect cells and transformed yeast cells. Though intracellular-form of virus-like particles or E protein aggregates was observed [16,32], they failed to secrete in these systems. In the present study, for the first time the above JEV cassette was expressed in the plasmid-based, nonviral, nonlytic Drosophila Expression System.

The coding sequence of signal-preM-E cassette used in this study was PCR-amplified from a full-length cDNA cosmid clone of JEV SA14 strain, pJEV-1. A polymerase with proof-reading activity was used in all PCR reactions to ensure faithful amplification. The sequencing data showed that pAc/ZF-ZR, pAc/JEF-JER and pJEV-1 had the identical sequence in the signal-preM-E region, though six differences were found when it was compared with the published sequence. It appeared that these differences had no adverse effect on the expression and antigenecity of E proteins in either S2 or Vero cells: the E protein was cleaved to the expected size and reactive with all three E protein specific MAbs, 2F2, 2H4 and mc3. All these three MAbs are neutralising *in vitro *and protective *in vivo*, and have been approved for phase-I and II clinical trials as a specific treatment agent for JEV infection in China. It is encouraging to find that the recombinant E protein produced in either S2 cells or Vero cells lost its reactivity with these MAbs in the presence of reducing reagents. This indicated that the E protein expressed in S2 cells was correctly folded to present authentic epitopes. These findings may explain previous observations that E protein produced in *E. coli *[33] and the authentic E protein prepared in denaturing conditions [34] failed to induce neutralising antibodies.

The E protein expressed in S2/ZF-ZR cells were found accumulated within vesicle-like structures in the cytoplasm, and also secreted into the culture supernatant. Interestingly, the secretion of E protein in S2/JEF-JER cells, which express shorter preM signal, preM and E, was dramatically reduced. This was consistent with the observation made with recombinant vaccinia viruses encoding the similar cassettes from other flaviviruses, that longer preM signal sequences led to higher secretion level of E proteins [17]. It was proved in the current study that the JEV preM signal peptide was recognized by *Drosophila *S2 cells and played an important role in the transportation and secretion of the E protein.

The E protein in the culture supernatant of S2/ZF-ZR was in a particulate form as shown in the sucrose density gradient centrifugation. The Triton X-100 treatment experiment further suggests that these particles were membrane vesicles with the entrapped antigens. We have not confirmed immunologically the presence of preM/M protein in the particles due to lack of appropriate antibodies. There have been no reports on the buoyant density of JEV EPs produced in mammalian cells [10,11]. In this study, S2/ZF-ZR-produced EPs migrated to the fraction with an average sucrose concentration of 34% (W/W) and a density of 1.13 g/ml, which are very close to that of other flavivirus-like particles expressed in mammalian cells [16] or yeast cells [32]. In agreement with the observation made with JEV EPs produced in the mammalian cells [11], the EPs produced by S2/ZF-ZR cells showed a much stronger immunogenicity in mice than the intracellular form of E protein within the cell lysate.

Another important feature of the S2/ZF-ZR cell line is its stability in the constitutive expression of JEV E protein. The yield of E protein remained at a similar level during two months of *in vitro *maintenance in the presence of Hygromycin B, indicating that the genotype of this cell line is stable and S2 cells can tolerate JEV E protein.

## Conclusion

JEV subviral extracellular particles containing functional E protein were expressed constitutively in a stable S2 cell line. Drosophila expression system is more convenient, cheaper and safer approach to the expression of proteins of interest, and may represent a promising alternative in the production of vaccine candidates and diagnostic reagents for JEV or other flaviviruses.

## Methods

### Construction of recombinant expression vectors

The coding sequence for 19-peptide preM signal peptide, preM and E proteins was amplified by PCR from a cosmid pJEV-1 containing the full-length cDNA of JEV SA14 [35]. The sense primer ZF, 5'-GC **GAA TTC ***ATG *GAA GGC TCA ATC CAT GTG G-3', was engineered with an Eco RI site (in bold) and a start codon (in italics) prior to the nucleotides (nt) 420–439 in the JEV genome. The antisense primer ZR, at nt position 2460–2478, 5'-GC **CTCGAG ***CTA *AGC ATG CAC ATT GGT CGC-3', has an engineered Xho I (in bold) and a stop codon (in italics). For comparison, the coding sequence for a shorter preM signal peptide (9-peptide), preM and E proteins was amplified with the primer pair JEF/JER (5'-C **GGTACC ***ATG *GCA GTT GTC TAG CTT GTG C-3'/5'- C **G AATTC **AGC ATG CAC ATT GGT CGC TAA G-3'). The PCR product should contain an engineered Kpn I site and start codon at the 5' end prior to nt 450, and an Eco RI site at the 3' end but no stop codon. The Expand Long Template PCR system (Roche) was employed in PCR amplification according to manufacturer's instructions. The PCR product primed by ZF/ZR were double digested with Eco RI/Xho I, purified with the Qiagen Gel Purification Kit (Qiagen) and ligated with the linearised DES constitutive expression vector pAc5.1/V5-His A or the mammalian expression vector pVAX1 (Invitrogen). The recombinant expression vectors were designated as pAc/ZF-ZR and pVAX/ZF-ZR respectively. The PCR product primed by JEF/JER was double digested with Kpn I/Eco RI, and ligated to appropriately linearised vector pAc5.1/V5-His A or pVAX1 to generate the recombinant plasmid, pAc/JEF-JER or pVAX/JEF-JER. The cloning sites and full-length inserts of these four recombinant plasmids were sequenced. Plasmid DNA was prepared using Qiagen Midiprep Kit (Qiagen).

### Sequence Analysis

DNA sequencing and data analysis were performed as previously described [36].

### Cell lines and antibodies

S2 cells were cultured in the DES Expression Medium (Invitrogen) supplemented with 10% heat-inactivated fetal bovine serum (Life Technologies) at room temperature without CO_2_. The DES Serum-Free Medium (Invitrogen) was used for expression and purification of secreted proteins. Vero cells were cultured in DMEM medium (Life Technologies) supplemented with 10% FBS and were incubated at 37°C in an atmosphere containing 5% CO_2_. Three monoclonal antibodies (MAbs) against JEV E protein, mc3, 2H4 and 2F2 were used in the detection of expressed E protein [37].

### Transfection of pAc/ZF-ZR or pAc/JEF-JER into S2 cells

The calcium phosphate coprecipitation method was used to produce stable transformants. The transfection of S2 cells cultured in a 35 mm dish was carried out with 20 μg of pAc/ZF-ZR or pAc/JEF-JER and 1 μg of pCoHYGRO (Invitrogen), a plasmid carrying the hygromycin B-resistance gene. The calcium phosphate-DNA precipitate was incubated with the cells for 16–24 hr. The cells were then washed twice gently with complete DES Expression Medium, resuspended in 3 ml of DES Expression Medium and replated into the original dish. After 2 days of further incubation, the cells were centrifuged and resuspended in complete medium containing hygromycin B (Invitrogen) at a concentration of 300 μg/ml. The selective medium was replaced every 4–5 days until resistant cells started growing out in about 3 weeks. These cells were designated as S2/ZF-ZR and S2/JEF-JER respectively.

### Transient transfection of Vero cells with pVAX/ZF-ZR or pVAX/JEF-JER

The monolayer of Vero cells in a 35 mm plate was transfected with 5 μg of pVAX/ZF-ZR or pVAX/JEF-JER in the presence of Lipofectamine (Life Technologies) according to the manufacturer's instruction. In parallel, the transfection was also carried out on 8-well chamber slides (Nunc).

### Immunofluorescence analysis

Transfected or non-transfected S2 cells were pelleted, resuspended in PBS and plated onto microscopic slides. After air drying, the cells were fixed in 10% formalin with 1% Triton X-100 at room temperature for 30 min. Transfected Vero cells grown on chamber slides were washed with PBS and fixed similarly. The cells were washed and incubated with a JEV E specific monoclonal antibody, and then with a fluorescein-conjugated secondary antibody (DAKO) at room temperature for 1 hr. Each incubation was followed by two washes in PBS. Finally the slides were mounted with VECTASHIELD Mounting Medium containing propidium iodide (Vector Laboratories) and viewed under an Olympus fluorescence microscope. Transfected S2 cells were further examined under a confocal microscope (Leiz).

### SDS-PAGE and Western blot analysis

Transfected S2 or Vero cells were lysed at 37°C for 15 min in the lysis buffer (50 mM Tris, pH 7.8, 150 mM NaCl, 1% Nonidet P-40) supplemented with protease inhibitor cocktail tablets (Roche). After centrifugation at 13,000 rpm for 15 min, the supernatant was recovered and stored at -20°C. For SDS-PAGE, the cell lysate or culture medium was mixed with 4 × sample buffer with or without β-mercaptoethanol, and boiled for 5 min. After electrophoresis on a 12% gel, protein bands were stained with Quick Blue or Silver Stain (Sigma). Proteins were also transferred to nitrocellulose membranes, probed with JEV E specific antibodies and visualized by ECL reagents (Amersham).

### Sucrose density gradient centrifugation analysis

S2/ZF-ZR cells (about 1 × 10^7^) maintained in complete DES Expression Medium with 300 μg of hygromycin B were pelleted to remove the selective medium. They were cultured for further 2 days in 10 ml of DES Serum-Free Medium before the medium was collected and clarified by centrifugation at 3,000 rpm. The clarified medium was then centrifuged for 15 hr at 25,000 rpm in a Sorvall TH641 rotor at 4°C onto a 10% sucrose cushion (W/V, prepared with PBS). The pellet was resuspended in 1 ml of PBS and applied to a 10–60% continuous sucrose gradient (W/V, prepared with PBS on BioComp automatic gradient former) and centrifuged for 12 hr at 36,000 rpm in a Sorvall TH641 rotor at 4°C. Fractions were collected by upward displacement with MAXIDENS (Lipotek). The sucrose density in the gradient fractions was determined with a handheld refractometer (Bellingam + Stanley Limited). All fractions were analysed by Western blotting with JEV E specific antibodies.

### Treatment with Triton X-100 (TX100)

Sucrose centrifugation-purified extracellular particles were diluted in PBS in the presence or absence of TX100 (final concentration of 1 mg/ml), and then applied to a 10–60% continuous sucrose gradient (W/V) prepared with or without 1 mg/ml TX100. Both gradients were centrifuged for 3 hr at 35,000 rpm in a Sorvall TH641 rotor at 4°C, and fractions were examined for JEV E antigenic reactivity by Western blotting.

### Animal experiments

Four-week-old female Balb/c mice were randomly grouped (4 animals per group) and immunized with PBS, S2/ZF-ZR lysate or extracellular particles containing E protein purified from the culture medium by sucrose density gradient centrifugation. Each mouse received about 10 μg E protein from cell lysate or 1 μg from sucrose fractions by both subcutaneous (s.c.) and intraperitoneal injection and (i.p.). For s.c. inoculation, the immunogen was emulsified with Freund's complete adjuvant, while no adjuvant was used for the i.p. route. All the mice were boosted with the same dose two weeks later. Blood samples were taken from tails two weeks after the boost and tested for JEV specific antibodies by Western blot. The investigation conforms to the Animals (Scientific Procedures) Act 1986, UK (Home Office Reference: PIL 70/16040). The cell lysate of pVAX/ZF-ZR-transfected Vero cells was used as the antigen.

## Competing interests

The author(s) declare that they have no competing interests.

## Authors' contributions

FZ designed the study, carried out the molecular biology and animal investigations, and drafted the manuscript. WM participated in the design and helped to draft the manuscript. LZ and MA participated in the animal study. HZ participated in study design, coordinated and supervised the study and finalised the manuscript. All authors read and approved the final manuscript.

## References

[B1] Solomon T (1997). Viral encephalitis in Southeast Asia. Neurol Infect Epidemiol.

[B2] Solomon T (2006). Control of Japanese encephalitis – within our grasp?. NEJM.

[B3] Paul WS, Moore PS, Karabatsos N, Flood SP, Yamada S, Jackson T, Tsai TF (1993). Outbreak of Japanese encephalitis on the island of Saipan. J Infect Dis.

[B4] Hanna JN, Ritchie SA, Phillips DA, Shield J, Bailey MC, Mackenzie JS, Poidinger M, McCall BJ, Mills PJ (1996). An outbreak of Japanese encephalitis in the Torres Strait, Australia, 1995. The Med J Austra.

[B5] Hanna JN, Ritchie SA, Phillips DA, Lee JM, Hills SL, van den Hurk AF, Pyke AT, Johansen CA, Mackenzie JS (1999). Japanese encephalitis in north Queensland, Australia, 1998. The Med J Austra.

[B6] Burdon JT, Stanley PJ, Lloyd G, Jones NC (1994). A case of Japanese encephalitis. J Infect.

[B7] Saito M, Sunagawa T, Makino Y, Tadano M, Hasegawa H, Kanemura K, Zamami Y, Killenbeck BJ, Fukunaga T (1999). Three Japanese encephalitis cases in Okinawa, Japan, 1991. Southeast Asian J Tropical Medicine Public Health.

[B8] Chambers TJ, McCourt DW, Rice CM (1990). Production of yellow fever virus proteins in infected cells: identification of discrete polyprotein species and analysis of cleavage kinetics using region-specific polyclonal antisera. Virology.

[B9] Russell PK, Brandt WE, Dalrymple JM, Schlesinger RW (1980). Chemical and antigenic structure of flaviviruses. The Togaviruses.

[B10] Mason PW, Pincus S, Fournier M, Mason TL, Shope RE, Paoletti E (1991). Japanese encephalitis virus-vaccinia recombinants produce particulate forms of the structural membrane proteins and induce high level of protection against lethal JEV infection. Virology.

[B11] Konishi E, Pincus S, Paoletti E, Shope RE, Burrage T, Mason PW (1992). Mice immunized with a subviral particle containing the Japanese encephalitis virus prM/M and E proteins are protected from lethal JEV infection. Virology.

[B12] Pincus S, Mason PW, Konishi E, Fonseca BA, Shope RE, Rice CM, Paoletti E (1992). Recombinant vaccinia virus producing the prM and E proteins of yellow fever virus protects mice from lethal yellow fever encephalitis. Virology.

[B13] Fonseca BA, Pincus S, Shope RE, Paoletti E, Mason PW (1994). Recombinant vaccinia viruses co-expressing dengue-1 glycoproteins prM and E induce neutralizing antibodies in mice. Vaccine.

[B14] Heinz FX, Allison SL, Stiasny K, Schalich J, Holzmann H, Mandl CW, Kunz C (1995). Recombinant and virion-derived soluble and particulate immunogens for vaccination against tick-borne encephalitis. Vaccine.

[B15] Pugachev KV, Mason PW, Shope RE, Frey TK (1995). Double-subgenomic Sindbis virus recombinants expressing immunogenic proteins of Japanese encephalitis virus induce significant protection in mice against lethal JEV infection. Virology.

[B16] Schalich J, Allison SL, Stiasny K, Mandl CW, Kunz C, Heinz FX (1996). Recombinant subviral particles from tick-borne encephalitis virus are fusogenic and provide a model system for studying flavivirus envelope glycoprotein functions. J Virol.

[B17] Konishi E, Pincus S, Fonseca BA, Shope RE, Paoletti E, Mason PW (1991). Comparison of protective immunity elicited by recombinant vaccinia viruses that synthesize E or NS1 of Japanese encephalitis virus. Virology.

[B18] Konishi E, Yamaoka M, Kurane I, Mason PW (2000). Japanese encephalitis DNA vaccine candidates expressing premembrane and envelope genes induce virus-specific memory B cells and long-lasting antibodies in swine. Virology.

[B19] Feng GH, Liu N, Zhou Y, Zhai YZ, Li XM, Dou XG (2006). Immunogenic analysis induced by DNA vaccine encoding E protein of Beijing-1 strain derived from Japanese encephalitis virus. Intervirology.

[B20] Konishi E, Mason PW, Shope RE (1996). Enzyme-linked immunosorbent assay using recombinant antigens for serodiagnosis of Japanese encephalitis. J Med Virol.

[B21] Matsuura Y, Miyamoto M, Sato T, Morita C, Yasui K (1989). Characterization of Japanese encephalitis virus envelope protein expressed by recombinant baculoviruses. Virology.

[B22] McCown J, Cochran M, Putnak R, Feighny R, Burrous J, Henchal E, Hoke C (1990). Protection of mice against lethal Japanese encephalitis with a recombinant baculovirus vaccine. Am J Trop Med Hyg.

[B23] Adams MD, Celniker SE, Holt RA (2000). The genome sequence of Drosophila meganogaster. Science.

[B24] Maroni G, Otto E, Lastowski-Perry D (1986). Molecular and cytogenetic characterization of a metallothionein gene of Drosophila. Genetics.

[B25] Bunch TA, Grinblat Y, Goldstein LS (1988). Characterization and use of the Drosophila Metallothionein promoter in cultured Drosophila melanogaster cells. Nucleic Acids Research.

[B26] Chung YT, Keller EB (1990). Positive and negative regulatory elements mediating transcription from the Drosophila melanogaster Actin 5C distal promoter. Mol Cell Biol.

[B27] Brighty DW, Rosenberg M, Chen IS, Ivey-Hoyle M (1991). Envelope proteins from clinical isolates of human immunodeficiency virus type 1 that are refractory to neutralization by soluble CD4 possess high affinity for the CD4 receptor. PNAS USA.

[B28] Lieberman MM, Clements DE, Ogata S, Wang G, Corpuz G, Wong T, Martyak T, Gilson L, Coller BA, Leung J, Watts DM, Tesh RB, Siirin M, Travassos da Rosa A, Humphreys T, Weeks-Levy C (2007). Preparation and immunogenic properties of a recombinant West Nile subunit vaccine. Vaccine.

[B29] Chambers TJ, Hahn CS, Galler R, Rice CM (1990). Flavivirus genome organization, expression and replication. Ann Rev Microbiol.

[B30] Konishi E, Mason PW (1993). Proper maturation of the Japanese encephalitis virus envelope glycoprotein requires cosynthesis with the premembrane protein. J Virol.

[B31] Konishi E, Win KS, Kurane I, Mason PW, Shope RE, Ennis FA (1997). Particulate vaccine candidate for Japanese encephalitis induces long-lasting virus-specific memory T lymphocytes in mice. Vaccine.

[B32] Sugrue RJ, Fu J, Howe J, Chan YC (1997). Expression of the Dengue virus structural proteins in Pichia pastoris leads to the generation of virus-like particles. J Gen Virol.

[B33] Mason PW (1989). Maturation of Japanese encephalitis virus glycoproteins produced by infected mammalian and mosquito cells. Virology.

[B34] Wengler G, Wengler G (1989). An analysis of the antibody response against West Nile virus E protein purified by SDS-PAGE indicates that this protein does not contain sequential epitopes for efficient induction of neutralizing antibodies. J Gen Virol.

[B35] Zhang F, Huang Q, Ma W, Jiang S, Fan Y, Zhang H (2001). Amplification and cloning of the full-length genome of Japanese encephalitis virus by a novel long RT-PCR protocol in a cosmid vector. J Virol Methods.

[B36] Zhang H, Morgan-Capner P, Latif N, Pandolfino YA, Fan W, Dunn MJ, Archard LC (1997). Characterization of stable attenuated variants that protect against infection with the cardiovirulent wild-type strain. Am J Path.

[B37] Zhang M, Wang M, Jiang S, Ma W (1989). Passive protection of mice, goats, and monkeys against Japanese encephalitis with monoclonal antibodies. J Med Virol.

